# Updates and Current Challenges in Reproductive Microbiome: A Comparative Analysis between Cows and Women

**DOI:** 10.3390/ani14131971

**Published:** 2024-07-03

**Authors:** Amanda Fonseca Zangirolamo, Anne Kemmer Souza, Deborah Nakayama Yokomizo, Ana Karolyne Alves Miguel, Márcio Carvalho da Costa, Amauri Alcindo Alfieri, Marcelo Marcondes Seneda

**Affiliations:** 1 National Institute of Science and Technology for Dairy Production Chain (INCT–LEITE), Universidade Estadual de Londrina, Londrina 86057-970, PR, Brazil; amandafz@uel.br (A.F.Z.); alfieri@uel.br (A.A.A.); 2 Laboratory of Animal Reproduction, Universidade Estadual de Londrina, Londrina 86057-970, PR, Brazil; aks.kemmer@gmail.com (A.K.S.); deborah.yokomizo@uel.br (D.N.Y.); ana.karolyne.alves@uel.br (A.K.A.M.); 3 Département de Biomédecine Vétérinaire, University of Montreal, Montreal, QC H3T 1J4, Canada; marcio.costa@umontreal.ca

**Keywords:** cattle, reproduction, vaginal microbiota, uterine microbiota, microbiota manipulation

## Abstract

**Simple Summary:**

We reviewed the intricate world of the reproductive microbiota in cattle, shedding light on its pivotal role in fertility. Through an extensive analysis of the current research, this article navigates through fundamental concepts such as microbiota composition, and interactions between pathogenic and non-pathogenic microorganisms within reproductive organs. We highlight the challenges involving the reproductive microbiota and their intricate associations with fertility outcomes. Furthermore, this review explores cutting-edge topics, including microbiota manipulation through innovative strategies and tools, offering insights into potential advancements in this field. Challenges and limitations facing the understanding and manipulation of the bovine reproductive tract microbiota are also discussed, alongside alternative approaches to propel the field forward. This article serves as a comprehensive resource for researchers, veterinarians, and stakeholders interested in enhancing fertility outcomes in cattle through microbiota management.

**Abstract:**

The microbiota plays an important role in numerous physiological processes, pathogenesis, development, and metabolism in different animal species. In humans, several studies have demonstrated an association between the vaginal microbiota and fertility rates, and even success in assisted reproduction techniques. In the context of cattle reproduction, although few studies have addressed the microbiota in a healthy state (which is not associated with diseases that affect the reproductive tract of cows), changes in its composition also seem to influence fertility. This review aims to explain the importance of the reproductive microbiota in female bovines and what is available in the literature regarding its possible role in increasing fertility. What are the challenges involved in this process? Future perspectives on its use and manipulation as a selection or intervention tool. Will it be possible to one day extrapolate the findings to reality and apply them in the field? In short, understanding the role of the reproductive microbiota of female bovines can signal the prospect of increasing production, whether of milk or meat, from the same number of animals, as it can optimize reproductive efficiency and perhaps become an allied tool for the economic profitability and sustainability of livestock farming.

## 1. Introduction

Previous studies have typically focused on microorganisms from a pathological perspective; however, our understanding of the host–microorganism relationship has changed from harmful to helpful and even essential. The microbiota plays an important role in numerous physiological processes, development, and metabolism of different animal species [1,2]. Therefore, the microbiome is considered a primordial component of the phenotype, as well as a potential complement to the host genome [3].

Microorganisms colonize almost all body components in a symbiotic relationship, primarily comprising specific bacterial populations based on the environment, age, sex, diet, body condition score, and phase of the estrous cycle [4]. Moreover, when there is an imbalance in these microbial communities, infertility, infections, functional changes, and other debilitating disorders can occur [5].

In humans, it is well reported that the vaginal microbiota interferes with women’s reproductive health, and several studies have demonstrated an association between the vaginal microbiota and fertility rates, and even with success in assisted reproduction techniques [6,7,8,9]. The vaginal microbiota has also demonstrated relevance to cattle reproduction, as changes in its composition seem to influence fertility [10]. Because of the growing demand for food and the importance of agriculture worldwide, an increase in actions and measures that can improve the reproductive performance of cattle is extremely important for the global economy.

Although several common reproductive disorders in animals involve bacterial infection, the characterization of a healthy vaginal and uterine microbiota of cattle is still at the beginning. Even when disease mechanisms seem to be misunderstood, microorganisms are considered important factors of influence and/or cause [3]. Therefore, to better understand the microbial potential in cattle reproduction, it is necessary to understand the characteristics and bacterial communities present in healthy and fertile females. Thus, the current challenge is to ensure the maintenance of beneficial bacterial communities to enhance reproduction and to control the dynamic interactions among all factors involved, whether internal or external to the animal.

In addition, understanding the reproductive tract microbiota is important for the application of measures aimed at improving the reproductive health of sows that have presented problems of unknown causes, especially subfertility and infertility [11]. Modulation of the vaginal microbial ecosystem has already been described in bovines as a measure that can reduce the indicators of infection in the genital tract [12,13]. Furthermore, tracking specific bacterial communities can also allow the identification of certain disease profiles in humans; thus, these bacteria can be used as biomarkers or even diagnostic tools [14].

Therefore, understanding the role of the reproductive microbiota in female bovines can signal the prospect of increasing production, whether milk or meat, as it can optimize reproductive efficiency and become increasingly essential for the economic profitability and sustainability of livestock farming. Finally, this review aims to explain the importance of the reproductive tract microbiota in cattle and describe the evidence available in the literature regarding its possible role in increasing fertility. Moreover, challenges and future perspectives regarding its use and manipulation as a selection or intervention tool in the field of reproduction in female bovines are discussed.

## 2. Understanding the Microbiota

The set of microorganisms that make up an ecosystem is called the microbiota, whereas the microorganisms, their metabolites, and their genetic material associated with a specific environment can be defined as a microbiome. Both are dynamic, changeable, and adaptable [15,16,17]. Although the microbiota is predominantly represented by bacteria, it also comprises archaea, protozoa, viruses, and fungi [18].

Microorganisms, specifically bacteria, must remain in balance with the host and other populations, favoring symbiosis [19]. Commensal bacteria maintain the integrity of the mucosa and control the proliferation of pathogenic microorganisms, either through competition for territory and food or through the production of enzymes that are toxic to these pathogens [15]. A small change in the composition of the bacterial community can trigger an imbalance between the microbiota and the host, and when there is a disproportionate proliferation of pathogenic agents, such an event is called dysbiosis [20]. In general, factors such as the use of antibiotics, illnesses, environment, and diet can influence the dynamics of the bacterial population and, consequently, the functioning of the microbiome [21].

Studies suggest that the human body contains as many microorganisms as human cells, and although the human genome is made up of 20 thousand genes, when all the genes present in its microbiome are combined, there are approximately 2 to 20 million genes [22]. Determination of the microbiota profile begins at birth, upon the first contact of the newborn with the maternal vaginal microbiota [23]. Although distributed throughout the body, most colonization occurs in the gastrointestinal tract, housing more than 100 trillion bacteria [24].

Several studies have described microbiota communities and their interactions with humans, animals, plants, and soil [25,26,27]. Thus, the identification of the microbiota and its influence on human and animal physiology has been the subject of research for numerous years, and the recognition of bacteria in the past was carried out only through cultivation. However, conventional culturing allows for the growth of only approximately 5% of the existing bacteria and underestimates the diversity of the microbiota analyzed [28].

To overcome this limitation, technological resources from 2005 onwards have been used as promising tools to understand the microbiome more deeply, in addition to becoming increasingly accessible [29]. Next-generation sequencing (NGS) techniques have been used to identify and characterize the diverse microbiota. Through NGS, it is also possible to classify microorganisms accurately and quickly as well as evaluate bacterial dynamics and their interactions with the environment [30,31,32]. Thus, the ultimate goal is to identify the species associated with reproductive health. 

## 3. Importance of the Microbiota

Several studies have demonstrated how bacteria would function in a synergistic commensal capacity and harmful pathogenic manner according to their location within the host. Therefore, the microbiota is fundamental to the health of the entire body in both animals and humans.

It is increasingly known that the microbiota can alter an individual’s health status and is related to a series of diseases, including reproductive disorders. Several studies have demonstrated that the maternal microbiome strongly influences the immune system development in newborns [33,34,35]. Dominguez-Bello et al. [23] observed a clear difference in the initial microbiota of newborns delivered vaginally or by cesarean section, and the initial microbiota seemed to have a significant impact on the individual’s future health. Vaginally born babies are naturally exposed to the microbiota present in the vagina of the mother during birth (predominantly *Lactobacillus*), which reduces the ability of pathogens to colonize. Babies born by cesarean section have skin microbiota that are more similar to the mother’s skin (*Staphylococcus*, *Corynebacterium*, and *Propionibacterium*), which are transmitted by the hospital staff with which the baby had contact and may justify the increased susceptibility to pathogenic bacteria [23]. 

Based on this information, there is an indication that the first natural colonization of bacteria throughout the newborn body occurs vertically [36]. After birth, the individual acquires a secondary microbiome through the environment and interactions with people, which is strongly influenced by the primary microbiome that controls the composition of future bacteria [37]. At 2 months of age, the number of species maintained in babies born vaginally was higher than in babies born through cesarean section [38]. However, regardless of the mode of transmission, the colonization of newborn microbiota is homogeneous in all habitats of the body. Therefore, the importance of the colonization of bacterial communities that positively impact neonatal development is great [23].

Since the period of microbiota acquisition coincides with the development of the immune system in a child, the bacteria transmitted to the baby early in life are crucial for providing an adequate immune response [39,40]. Even small environmental changes can modulate the composition of the lung microbiota early in life, and adults then show a greater resistance to environmental variations [41]. Similarly, in the ruminal microbiota observed in a previous study, diversity and similarity within the group increased with age, demonstrating a more diverse yet homogeneous and specific mature community compared to the less diverse and more heterogeneous primary community. Furthermore, convergence towards a mature bacterial arrangement with age was observed. These findings have also been reported for human gut microbiota [42].

Several studies have described intestinal bacterial communities and their interactions with humans, animals, and health in general [17,43]. Dysbiosis or changes in the microbiota can increase intestinal permeability, inflammation, and autoantibody formation [44]. Changes in immunity [45], autoimmune (immune-mediated) diseases [46], neurodegenerative diseases [47], respiratory diseases associated with allergies and asthma [48,49], in addition to obesity and diabetes [50,51,52], mainly in humans, have been reported as a response to intestinal microbiota imbalance. Furthermore, a reduced diversity of the intestinal microbiota may be associated with depressive symptoms, anxiety, and eating disorders [44].

Also, the clear relationship between the intestinal microbiota and health has encouraged subsequent research focused on the health of the vaginal microbiota in women and its possible link to infertility and neonatal issues [53]. In cattle, investigations have been conducted to improve feed efficiency through the manipulation of the intestinal microbiota [54,55,56] and the inhibition of bovine respiratory disease (BRD) [57,58]. This, in turn, can directly influence existing knowledge regarding the vaginal microbiota, as the authors have demonstrated a clear relationship between the intestinal microbiota and the composition of bacteria in the vagina [59].

The microbiota in niches, such as the uterus and vagina (mainly), have been associated by several authors with reproductive disorders and the failure of reproductive biotechniques. In humans, one in seven couples is unable to become pregnant even after 1 year of normal, unprotected sexual intercourse, with 35% of couples having an unknown cause [60]. One explanation for this is that infertile women have a different microbiota than fertile women [61,62,63].

Regarding the efficiency of in vitro fertilization treatments in women, a meta-analysis showed that patients who had an abnormal vaginal microbiota were approximately 1.4-fold less likely to become pregnant after the procedure than women with normal microbiota, demonstrating a strong correlation between an unbalanced vaginal microbiota and early pregnancy or in vitro fertilization failure [8]. Studies have shown that vaginal *Lactobacillus* can have a positive correlation with pregnancy rate [9] and is reduced in women who repeatedly fail to become pregnant [8].

Bacterial vaginosis (BV), characterized by increased bacterial diversity and associated with dysbiosis, is the most common vaginal disorder, affecting 10–50% of the women worldwide [64,65,66]. In a population of approximately 1200 women, one study related this bacterial pathology to fertility and observed a higher prevalence of the disease in women with infertility (45.5%) than in fertile women (15.4%) [67]. BV has been reported to increase the risk of miscarriage and premature birth in pregnant women by up to 2-fold compared to those with healthy vaginal microbiota [8,68]. Furthermore, BV is associated with pelvic inflammation, post-abortion sepsis, post-hysterectomy vaginal infections, and the acquisition of sexually transmitted diseases, such as gonorrhea, chlamydiosis, trichomoniasis, and human immunodeficiency virus (HIV) [7,69].

BV occurs due to a disruption of the vaginal balance and excessive growth of typically non-*Lactobacillus* and pathogenic anaerobic bacteria. In human females with healthy vaginal microbiota, the predominance of *Lactobacillus* is a sign of reproductive health. *Lactobacillus* and its main metabolites allow the vaginal homeostasis of women by maintaining an acidic environment (pH < 4.5) to protect it from infection by pathogens [70,71]. By producing lactic acid, *Lactobacillus* lowers the pH of the vaginal environment and protects the vagina from invasion and infection by opportunistic pathogens. In addition, several of these bacteria produce hydrogen peroxide, bacteriocins, glycogen, and glycerol, which ensure their survival and habitation in the reproductive tract [8].

Romero et al. [72] characterized the structure of the microbial community present in the vagina of pregnant women and found that throughout pregnancy, there was a dominance of *Lactobacillus* spp., suggesting that this bacterium is related to the stability of the microbiota and prevention of ascending infections that are linked to premature birth. Another study demonstrated that a lower abundance of *Lactobacillus* in pregnant women is associated with late miscarriages or premature births [6].

However, despite the potential importance of vaginal *Lactobacillus* in reducing the risk of reproductive disorders, an interesting fact is that in cattle, as well as in other species (sheep and non-human primates), *Lactobacillus* is found in small quantities and does not assume the same relevance as in humans [70,73,74].

## 4. Reproductive Microbiome in Cows: Pathogenic Interactions

Dysbiosis is a critical factor in various reproductive diseases. Furthermore, different research groups have analyzed the possibility of pathogenic bacterial strains that can lead to pregnancy failure. In this context, the infectious agents identified in the vagina of cows have already been associated with metritis, endometritis, infertility, placentitis, abortion, premature birth, and the birth of debilitated calves [75,76,77].

Studies have reported bacteria belonging to the gastrointestinal system of cattle in the vaginal microbiota and that a large proportion of reproductive disorders are caused by microorganisms found in the fecal material [78,79]. Therefore, it is worth highlighting that, in bovine species, the anatomical conformation of the anus and vagina means that the vulva is generally covered in feces, especially in females that have a horizontal vulva that forms an angle greater than 45° with a vertical plane. This condition allows the colonization of the vaginal canal by microorganisms from the intestinal microbiota in the early stages of life [80,81].

Therefore, the microbiota that predisposes individuals to reproductive disorders may be caused by the colonization of microorganisms that are not part of the normal vaginal community, resulting in non-ideal bacterial profiles, or by an increase in the virulence of commensal species [82,83]. Thus, compositional changes in the vaginal microbiota do not necessarily imply disease or result in clinical signs but might be a result of the interaction between microbial virulence and the host’s innate and adaptive immune responses [84]. 

While a healthy vaginal microbiota is protective against the colonization of pathogenic species, it has been suggested in humans and cattle that a large pathogenic bacterial load in the vagina can contaminate the rest of the female reproductive system in an ascending manner. These organisms may even reach the ovaries, negatively impacting follicular development by inhibiting the gonadotropin response [85,86]. Vaginal bacteria that ascend to the uterus can cause endometritis and have been detected more frequently in animals with clinical endometritis than in those without endometritis [86]. Negative changes in the vaginal microbiota, especially after parturition, are associated with uterine diseases in dairy cows [77].

Miranda-CasoLuengo et al. [87] were the first to compare the vaginal and uterine microbiota of black and white Dutch cattle and found that despite the great differences, there is a core community shared between the two organs. This similarity occurs mainly in the postpartum period, justified by the cervical opening during the birth of the calves, resulting in the dispersion and mixing of the microbiota. Excessive bacterial growth in the uterus after birth is considered normal. However, females who cannot control these bacteria within 21 days develop endometritis [87]. Thus, the explanation for the occurrence of postpartum endometritis in almost 90% of cows [88] is the contamination of the reproductive tract by microorganisms found in their feces because of the proximity of the anus to the vagina [81,89,90,91].

Wang et al. [86] evaluated and characterized the vaginal microbiota of healthy postpartum cows, relative to postpartum cows with endometritis, and demonstrated a significant difference between the vaginal bacterial communities of the two groups. They also suggested that alteration of the normal vaginal microbiota may contribute to the initiation of endometritis and that bacterial diversity in these females was significantly higher than in healthy cows, as the microbial community structure governed by the dominant bacteria was disrupted and the number of pathogenic bacteria increased. In addition, in a study conducted by Bicalho et al. [92] during the transition period (−7, 0, 3, and 7 days after birth), differences were observed in the bacterial composition of the vaginal microbiota and total bacterial load (TBL) related to disease and fertility. Microbiota composition and TBL are associated with known periparturient risk factors of uterine disease, reproductive failure, assisted birth, and retained fetal membranes [92]. 

The most common pathogens associated with endometritis are *Escherichia coli*, *Trueperella pyogenes*, *Prevotella melaninogenicus*, and *Fusobacterium necrophorum* [89], with *E. coli* being the most prevalent in cows with metritis during the first week postpartum [87]. Furthermore, bacteria belonging to the Porphyromonadaceae, Fusobacteriaceae, and Leptotrichiaceae families were associated with uterine disease in a study that followed cows from birth to the postpartum period, and that also showed that in the first evaluation before birth, bacterial signaling endometritis was already present [93]. Corroborating the above results, Wang et al. [27] described the Firmicutes phylum as predominant in the cervix of dairy cows. Cervical bacterial diversity was found to decrease in cows with metritis, and the predominant bacterial genera were *Porphyromonas* and *Fusobacterium* spp.

In a previous study, Wang et al. [86] compared the vaginal microbiota of healthy cows and cows with endometritis and observed that in healthy females, there was a dominance of *Lactobacillus sakei* and *Weissella koreensis*. Furthermore, *Histophilus* [19], *Ureaplasma* [79], *Tenericutes*, and Acidobacteria [10] were more abundant in cows with difficulty conceiving or in females with reproductive problems. Additionally, the dominance of the phyla Bacteroidetes and Fusobacteria has been observed in the vagina of cows with metritis [94,95]. 

In short, when considering the microbiota of the genital tract, it is possible to expand the understanding of reproductive failure, as changes in the composition of the bacterial community can modify the balance of commensal agents and predispose individuals to infection [19]. Another aspect of the study of the microbiota is the identification of healthy communities that could increase reproductive efficiency.

## 5. Reproductive Tract Microbiome in Cows: Non-Pathogenic Interactions and Association with Fertility

Under normal conditions, the vaginal microbiota has a variable composition and number, and the microorganisms found are also present on the skin and feces and may even be present in small numbers in the uterus of healthy cows. 

In this context, a mutualistic relationship between the host and the microorganisms that inhabit the vagina establishes the first line of defense against pathogenic colonization of the vaginal mucosa [82,96]. One immunological barrier is the formation of a biofilm by bacteria associated with the vaginal mucus, which increases the survival of the resident microbiota and consequently helps maintain the microbial environment during the estrous cycle and pregnancy phases [73]. Furthermore, these bacteria produce reactive oxygen species (ROS) and organic acids to inhibit infection by the main pathogens during the follicular phase of the estrous cycle [97].

When healthy, the bacterial community is stable; however, it can be affected by numerous factors, such as the environment, food, age, phase of the estrous cycle, pregnancy, management, animal genotype, and immunological response in humans and livestock [81,98,99]. Racial variation and the geographic area where women live are important, as they cause them to present relevant differences in the dominant vaginal microbiota [100]. Therefore, multiple variables affect microbiota, complicating attempts to identify clear patterns. External influences, such as diet and environment, along with physiological changes due to hormonal fluctuations, pH, stress, or illness, can affect and modify the composition of the microbiota. Bacterial communities are in a constant state of flux because several generations of a specific genus can evolve over a few days or even a few hours. Therefore, more frequent studies at different key times are crucial to understanding microbiota fluctuations.

Although the structure of the communities may differ between animals, it is possible for the health of the vagina and female reproductive system to be maintained as a whole, as long as the beneficial function of the bacteria is present. Knowledge of the composition of the vaginal and/or uterine microbiota has been limited until a few years ago, as there have only been qualitative descriptive studies using culture-dependent techniques [100].

Over the years, the most common microorganisms isolated from the vaginas of cows and identified by culture have been *Enterococcus* spp., *Staphylococcus* spp., and *Streptococcus* spp. [88,101,102,103]. However, the results obtained using this technique have already demonstrated that they are not sufficiently accurate to reflect the real microbial diversity of the samples. Thus, with the development of DNA sequencing, unprecedented information regarding the profiles of the bacterial communities related to health and reproductive diseases has been revealed. Swartz et al. [73] were the first to perform culture-independent 16S ribosomal RNA (rRNA) gene sequencing of the vaginal microbiota in cows and sheep. In this study, it was observed that there was a greater diversity of bacteria in the vaginal ecosystem than previously known, and the importance of the most abundant microorganisms within each community was highlighted.

**Figure 1:**
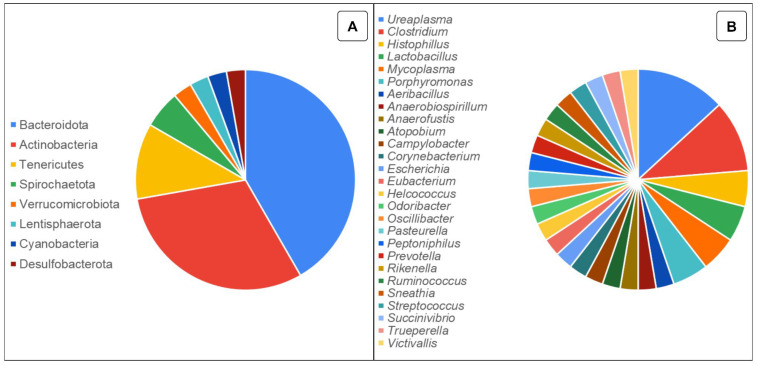
Representation of bacterial phyla (**A**) and genera (**B**) reported in the vaginal microbiota in healthy female cattle. The data do not represent relative abundance; instead, the number of articles/studies found reporting the presence of the same genus and phylum are shown.

In addition, it has become possible to compare the vaginal microbiota of these species with that of other species, such as humans, non-human primates, and pandas [74,104,105]. Then, it was observed that the microbiota of the bovine reproductive system presents greater diversity compared to the human vaginal microbiota [79]. In Figure 1, some phyla and genera of bacteria present in the vaginal microbiota already reported in healthy female cattle by various studies are described [106,107,108,109], among others cited in this review. In Supplementary Table S1, there is a list of authors/studies and their respective findings according to the uterine microbiota of healthy female cattle.

Although most published data on the vaginal microbiome have been derived from women, promising research has been conducted on cattle. A standard microbiota has not yet been defined because it is known to be influenced by several factors. However, recent studies have demonstrated that the most frequently detected bacterial phyla in the vaginas of dairy cattle are Firmicutes, Bacteroidetes, and Proteobacteria [81,96,110]. Quereda et al. [79] observed that in dairy cows, the phyla Tenericutes (35.6%), Firmicutes (25.2%), and Bacteroidetes (14.9%) represented more than 75% of the total vaginal microbiota. Other abundant phyla, such as Proteobacteria, Actinobacteria, Fusobacteria, Epsilonbacteraeota, and Patescibacteria, together with those mentioned above, represented more than 96% of the bacteria. In the present study, *Ureaplasma*, *Histophilus,* f_Corynebacteriaceae, *Porphyromonas*, *Mycoplasma*, and *Ruminococcaceae* UCG-005 were the most abundant genera.

**Figure 2:**
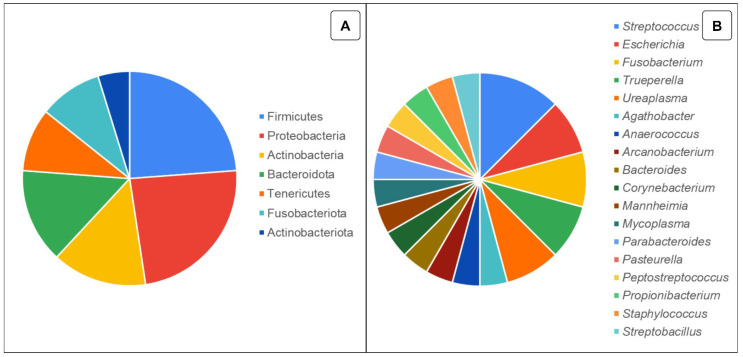
Representation of bacterial phyla (**A**) and genera (**B**) reported in the uterine microbiota in healthy female cattle. The data do not represent relative abundance; instead, the number of articles and studies found reporting the presence of the same genus and phylum are shown.

Importantly, bacteria described as abundant in healthy cows, such as Bacteroides (28.3%) and Enterobacteriaceae (17.8%), may have their relative abundance altered in reproductive disorders (35.8% and 18.6%, respectively) [19]. Therefore, it is necessary to understand the fluctuations in the vaginal microbiota according to the influence of the health and reproductive status of animals. In Figure 2, some phyla and genera of bacteria present in the uterine microbiota reported in healthy female cattle by several studies cited in this review are described. In Supplementary Table S2, the authors and studies and their respective findings are listed based on the uterine microbiota of healthy female cattle.

### Microbiota and Hormones

Fluctuations in the vaginal bacterial population are dependent on circulating steroid hormones [96], and the vaginal bacterial abundance in cows and sheep differs according to the stage of the estrous cycle [102,111]. Bovine females tend to have less abundant bacterial microbiota during the cycle, as characterized by the release of progesterone, and the same occurs during pregnancy and after birth. With the return to the normal estrous cycle, the vaginal microbial population tends to return to balance, owing to an increase in the bacterial population [81]. It has been observed that an increase in Firmicutes in the vagina is mainly due to a decrease in progesterone concentration. In contrast, the relative abundance of Proteobacteria is associated with an increase in the same hormone [10]. In addition, the relationship between progesterone and bacterial abundance explains the decrease in bacterial diversity within the uterus during the synchronization protocol, which is performed before fixed-time artificial insemination (FTAI) [10].

In contrast, estrogen decreases bacterial virulence, increases the growth rate of commensal bacteria in the community, and is highly correlated with high bacterial diversity [81]. Despite the low abundance of *Lactobacillus* spp. throughout the estrous cycle, they are found in greater quantities in the follicular phase (estrus) than in the luteal phase (diestrus) in bovine females [79]. These data corroborate human studies reporting that the *Lactobacillus* genus increases under estrogen’s influence and helps improve women’s conception [112]. In this context, primiparous cows have lower bacterial diversity than multiparous cows because of the exposure of the vagina to bacteria and the trauma resulting from the passage of the calf during birth in multiparous cows [11,113].

In women, it has been described that the vaginal microbiota remains more stable during pregnancy [72,114] since there is a maturation in the vaginal epithelium due to the increase in circulating estrogen levels produced by the placenta and the establishment of glycogen accumulation [115]. In turn, glycogen is broken down by the host α-amylase in the vaginal epithelium to make products that support the colonization of *Lactobacillus* spp. This genus is well presented in pregnant women. In the vaginal tract of healthy pregnant cows, *Lactobacillus* spp., *Pediococcus* spp., *Leuconostoc* spp., *Weissella* spp., Enterobacteriaceae, *E. coli*, and bacilli were identified with a greater predominance [116]. In bovine species, the vaginal microbiome of pregnant females is different from that of non-pregnant females, as the diversity of the microbiota decreases significantly during the luteal phase, when the reproductive tract is preparing for pregnancy [81,96].

Interestingly, commensal populations of the vaginal microbiota influence the odor profile of hosts, either through the production of odorants or through the metabolism of existing endogenous organic compounds [96,117]. Thus, the microbiota provides imperceptible communication between individuals of the same species, influencing the social, physiological, and sexual behaviors of animals [118,119].

## 6. Microbiota Manipulation: Strategies and Tools

Owing to the growing number of publications linking dysbiosis and pathogens with poor pregnancy rates and failed pregnancies, research has advocated the use of supplementation as a type of microbiota manipulation for multiple uses. In this context, most microbiota manipulation strategies involve the enrichment of the native species through supplementation with beneficial bacteria, which helps correct dysbiosis. The most used methods for manipulating the vaginal microbiota are probiotics. Another important method of manipulation is the inhibition of pathogenic bacteria using antibiotics.

Several probiotic and prebiotic supplementation regimens are commercially available. However, most manipulation strategies have focused on intestinal microbiota. A few studies have investigated these findings, applied them to vaginal microbiota, and observed improvements in the reproductive health of cows [120]. Therefore, improving reproductive health and, perhaps, pregnancy rates through any manipulation, whether through the use of prebiotics, probiotics, or even extrapolation to a possible microbiota transplant, is still at an early stage. Although research on the composition of healthy vaginal microbiota, even in humans, is still relatively limited, developing a reliable strategy could be the key to improving fertility rates in cows while maintaining a healthy microbiota.

**Figure 3:**
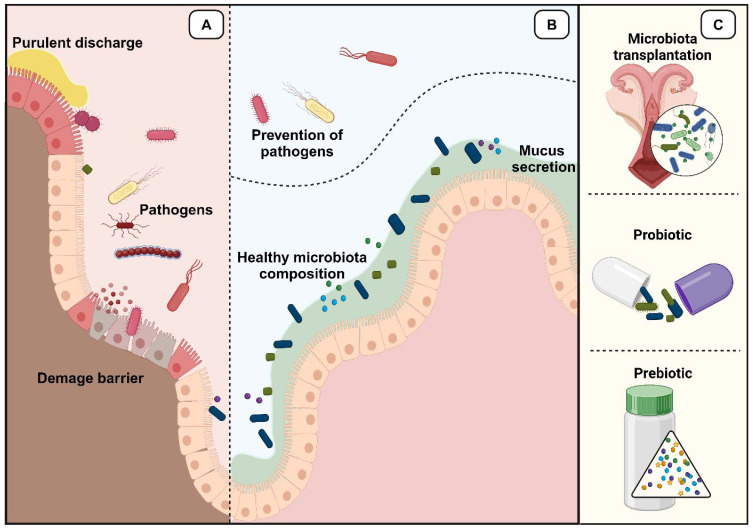
(**A**) Representation of the uterine/vaginal environment in the absence of healthy microbiota. (**B**) Uterine/vaginal environment in the presence of healthy microbiota, which prevents the development of diseases. (**C**) Possible tools to improve the composition of healthy uterine/vaginal microbiota.

Next, we explain some strategies and tools for manipulating the microbiota (Figure 3), with the first subtopic briefly reporting on important considerations regarding the frequent use of indiscriminate antibiotics. In the following subtopics, tools (such as prebiotics, probiotics, and microbiota transplantations) are presented that, if better investigated in the field of reproduction, could help optimize fertility and reproductive health results in female bovines.

### 6.1. Antibiotics

Although antibiotics have saved an incalculable number of lives since their discovery at the beginning of the 20th century, their indiscriminate use can increase the prevalence of resistant bacteria [121]. The association between antibiotic consumption and resistance has been well documented [122]. Antibiotics alter the intestinal microbiota, and studies have shown that changes in bacterial composition can be definitive or recovered over the long term [123,124,125].

Francino [126] reported that antibiotics are essential in treating diseases and infections, but they have been associated with adverse effects on intestinal microbiota. Antibiotics are primarily non-selective and can destroy many beneficial and pathogenic bacteria. Thus, excessive and indiscriminate use of antibiotics causes dysbiosis by reducing the diversity of the microbiota (an impact already well-studied in the intestine). This, in turn, negatively affects the host’s overall health and immune system. The effects of antibiotic use, especially when indiscriminate, have been described as causing or aggravating various diseases, such as inflammatory bowel disease, asthma, rheumatoid arthritis, diabetes, obesity, depression, autism, and even superinfection in seriously ill patients [127].

Furthermore, the use of antibiotics also affects the microbiota of the environment and the surrounding population, including the transmission of antibiotic-resistant bacteria [128]. This issue warns us that antibiotics should be more cautiously prescribed. Furthermore, there has been an increase in chronic and autoimmune diseases, and researchers have suggested that antibiotics may play a significant role by changing the bacterial populations that benefit the body [35].

In dairy farming, in addition to altering a healthy microbiota, the use of antibiotics leads to economic disadvantages because of increased production costs, loss of milk due to medicinal residues, development of microbial resistance to antibacterial drugs, adverse effects on the uterine epithelium, and myometrial contractility [120,129,130,131]. Therefore, one of the factors of great importance in fully understanding the role of the microbiota is to develop ways to enhance commensal bacteria with the expectation that the organism, in good functioning, can spontaneously recover [132].

### 6.2. Prebiotics

The definition of prebiotics established at the sixth Meeting of the International Scientific Association of Probiotics and Prebiotics (ISAPP) in 2008 and used to this day is “a selectively fermented ingredient that results in specific changes in the composition and/or activity of the gastrointestinal microbiota, thus conferring benefits on host health” [133]. Therefore, these nutrients present in food are degraded by the intestinal microbiota and promote improvements in the host’s health through the growth of beneficial microorganisms such as bacteria.

Prebiotics have some criteria, such as being resistant to the stomach’s acidic pH, not being hydrolyzed by mammalian enzymes, and not being absorbed in the gastrointestinal tract [133]. However, intestinal microbiota can ferment prebiotics, and their degradation results in short-chain fatty acids. These fatty acids are the primary energy source for colon cells, promoting intestinal health and generating several benefits. Among the benefits already reported is the reduction in diseases such as obesity, diabetes, and fatty liver disease, among others related to diet; moreover, it also helps with calcium absorption, keratin retention, and collagen formation [134].

A study involving lactoferrin (prebiotic) in cattle related to a direct prebiotic activity by stimulating the growth of specific probiotics at 22 °C. However, despite the numerous advantages, including the ease of being provided through the addition of diets rich in fiber and grains, there is a need for more research to study the possible links between the maintenance of a healthy vaginal microbiota through supplementation with prebiotics to improve the reproductive health in cattle.

### 6.3. Probiotics

Probiotics are live microorganisms that provide health benefits to the host when administered in adequate quantities (World Health Organization, 2001). Owing to their safe and natural characteristics, probiotics are currently considered treatment alternatives with the advantage of establishing microbial homeostasis in the female reproductive tract [44,135].

Dietary supplements with live agents promote health by stimulating the growth of commensal microorganisms, reducing the number of pathogenic or potentially harmful bacteria, and reinforcing immune mechanisms [136]. Although the extent to which probiotics influence the microbiota has not yet been determined, promising results have been reported, particularly in humans. To date, most of the probiotic species studied include *Lactobacillus* (*L. casei*, *L. fermentum*, *L. plantarum*, *L. salivarius*, *L. acidophilus*, *L. paracasei*, *L. reuteri*, and *L. rhamnosus*) and the genera *Bifidobacterium* (*B. breve*, *B. lactis*, *B. thermophilum*, *B. bifidum*, *B. infantis*, and *B. longum*) [135,137,138].

The treatment of BV with antibiotics is standard in women [139]. However, failure to cure, disease recurrence, and the emergence of antibiotic resistance have been widely observed [140,141,142]. Therefore, probiotics have been used as novel therapeutic agents. Kyono et al. [143] stated that a high level of *Lactobacilli* may improve the implantation rate, especially in women undergoing in vitro fertilization, once again addressing the advantage of using probiotics in an attempt to re-establish vaginal eubiosis to enhance reproduction [135]. Notably, the bovine vaginal microbiota carries a different composition than that of humans, and more species-specific strains of bacteria should be considered.

In this context, the use of these bioactive components has also been described in the control of gestational disorders in women, such as glucose intolerance [144,145] and dyslipidemia [138], as well as in the prevention of premature labor [146]. Furthermore, modulating the maternal microbiota through probiotic interventions is a generally safe approach with the potential to recover the commensal community and provide advantages to the health of the mother, fetus, and baby [147,148].

In dairy cattle, probiotics can improve reproduction by reducing the administration of antibiotics during postpartum infection and endometritis, which inhibit the uterine immune function and act as irritating factors [88,149]. Moreover, it has been reported that probiotics reduce the indicators of postpartum uterine infection and assist in the efficiency of repair and involution of the uterus by modulating the vaginal microbiota and consequently preventing the growth of pathogenic bacteria [12]. In addition, milk from cows treated with antibiotics can only be consumed after the withdrawal period.

Given the potential of lactobacilli and other acidifying bacteria in maintaining a healthy gut and vaginal microbiota in humans, researchers have attempted to develop similar probiotics for use in cattle. Otero and Nader-Macıas [150] used heifers (Nellore–Hereford, and Criolla) to identify vaginal microbiota through culture. That study aimed to formulate probiotic products for veterinary applications to prevent infectious diseases by restoring the microbiota, reducing the need for antibiotic and hormonal treatments. The authors concluded that the use of *Lactobacilli* in the vagina of cattle could be an alternative to prevent metritis and improve the reproductive performance of cattle.

Elevated levels of these bacteria during the estrus phase would coincide with the increase in estrogen in estrus, as seen in the hormonal study by Parish et al. [151], since increased estrogen levels inhibit the growth of several bacteria thus allowing a select few to remain. In a previous study, Otero et al. [102] developed a probiotic for veterinary use in cows to establish an optimal vaginal microbiota. Samples were collected during the proestrus, estrus, metestrus, and diestrus phases of two cycles from 15 Nellore Hereford heifers. *Lactobacilli* and *Enterococci* were present in low numbers in all three phases, whereas their numbers increased slightly in the estrus phase. However, *Enterococci* bacteria counts were significantly higher than that of *Lactobacilli* (102 and 104 CFU/sample, respectively) throughout the cycle [102].

However, despite its demonstrated efficiency, a limiting factor is the lack of consideration regarding the variety in the general composition of the vaginal microbiota, with *Lactobacillus* being one of the only agents. Furthermore, research has shown that the indiscriminate and prolonged use of probiotics can cause metabolic changes [152]. In young animals, excessive use of probiotic supplements during the early stages of life might disrupt the establishment of a healthy intestinal microbiota [153]. Therefore, caution is recommended when using these tools, particularly for healthy cows and calves.

It should be mentioned that most studies evaluating the impact of probiotics on cows used a small number of animals. Further, larger cohorts are necessary before sound conclusions about this strategy can be made. Thus, more research is needed on the development of new probiotic formulas specific to each need, particularly in veterinary medicine.

### 6.4. Microbiota Transplantation

Microbiota transplantation is a recently developed tool for transferring bacteria to a desired environment. This procedure is performed from a healthy donor to another individual (recipient) who presents with dysbiosis or pathological changes to restore the balance of the microbiota and help control diseases [154]. This practice is best established in the intestinal microbiota, as the intestinal tract is home to one of the richest and most complex microbial populations and plays a critical role in health and a wide range of diseases.

In humans, fecal microbiota transplantation (FMT) has achieved great success in treating intestinal infections (i.e., *Clostridioides difficile* infection), especially those related to antibiotic-resistant pathogens [155]. In addition, cures or improvements after the use of FMT have been reported in general infectious diseases, inflammatory bowel disease, metabolic diseases such as obesity/diabetes, cardiovascular diseases, hepatitis, mental illnesses (such as depression), neurological disorders (such as autism, Parkinson’s disease, and Alzheimer’s disease), and immune system diseases [154]. FMT has also been shown to interact with immune cell infiltrates, gene expression profiles in the tumor microenvironment [156,157,158], and the expression of markers of innate immune activation and activation of immune cells [159,160]. However, it is worth noting that the frequency and dose of FMT can affect the therapy results [154].

The biological safety of FMT has always been a concern and has attracted increasing attention in recent years. According to the US Food and Drug Administration (FDA, 2020), cross-infection by pathogenic bacteria has previously occurred, likely induced by inadequate donor selection and biological tests or by non-standardized processing procedures. To ensure the technique’s safety, stool banks have been established, alternative FMT material preparation processes, such as washed microbiota transplantation, have been developed, and products have been approved under strict standards [161]. However, models related to donor selection, supervision systems, and FMT procedures have yet to be unified worldwide [162].

From a potential perspective, the transferred fecal microbiota could restore the intestinal microbiota of the recipient animal and thus improve reproductive function because of the close anatomical relationship between the anus and vagina. In this context, it is also worth remembering that reproductive disorders are chiefly caused by microorganisms found in fecal material [79,81]. Therefore, microbiota transplant treatment is expected to regulate the vaginal and uterine microbiota with minimal damage to animals and restore the reproductive health of recipient females through healthy microbiota from donors.

Although there are no studies in cows, another form of microbial transfer therapy is vaginal microbiota transplantation (VMT), which involves transferring vaginal microbiota from a healthy female donor to a diseased female vaginal cavity that aims to restore the otherwise imbalanced vaginal microbiota [163]. VMT has been applied in the treatment of bacterial vaginosis (BV), obtaining satisfactory results and without any adverse effects in women [25]. In this context, it has also been reported that applying Synthetic Bacterial Consortia Transplantation (SBCT) and VMT treatment decreased the bacterial load of *Gardnerella vaginalis* in the vaginal region of mice afflicted with BV [164]. Furthermore, recently, Wrønding and collaborators [165] reported a successful VMT, with a subsequent successful pregnancy and birth after several episodes of late pregnancy losses/stillbirths in women. However, the potential of VMT to improve women’s health is still in its early stages of development and requires extensive research not only for humans but also involving other species, such as cattle [166].

In short, more investigations and research are needed to evaluate and compare whether FMT produces promising results in the reproductive microbiota or whether, similar to the intestinal microbiota, it is possible to transplant healthy vaginal microbiota into sick animals directly. Thus, the possibility of exploring the ideal reproductive microbiota to optimize cattle reproduction, finding more suitable donors, and performing targeted and efficient treatments is expected.

## 7. Challenges

Based on the discussion and reports in this review, it is clear that ensuring the maintenance or growth of beneficial bacterial communities to enhance reproduction and control the dynamic interactions among all the factors involved is of paramount importance. Therefore, further studies are required to isolate and track bacteria from the vaginal tract of healthy cows that are beneficial for reproduction in order to truly understand the effects of pre- and probiotics on the microbiota. Establishing and exploring alternatives to microbiota transplantation is necessary to optimize the reproduction of animals with dysbiosis.

Furthermore, knowledge of factors such as the dose, route, and method of administration, single or multiple strains of bacteria, and standardized methods of measuring the microbiota are relevant for developing specific actions and tools for manipulating the microbiota in cattle and enhancing reproduction.

In addition, NGS methodologies depend on bioinformatics and a personalized data library to assist in analyzing and interpreting the obtained data [31]. The information regarding bacterial communities identified by NGS presents high complexity and variability, and the need for standards and protocols for comparing results at the global level is a limiting factor. At each stage of the process, from the laboratory environment to the DNA extraction and amplification kits, there may be changes in the results of the microbiota analysis, especially in low-biomass samples [167]. Another obstacle in most NGSs is the high cost per sample analyzed and the need for professionals with specialized training to analyze the data [31].

A limitation of several sequencing techniques is that they do not reach the species level, making it impractical to accurately compare the identified genome due to incorrect DNA base insertions during the process thus needing better resolution [15]. Another challenge encountered in NGS studies is the inability of the databases to recognize the identified genetic material, either because of outdated libraries or the difficulty in detecting the strain due to the distance that the new genes have from the already known species [15,168]. The accuracy of species identification using databases also requires the presence of the correct taxonomies, but numerous microorganisms still need to be discovered or have yet to be genetically mapped [169].

Souza et al. [169] concluded that even long-read sequencing technologies (PacBio), which should classify bacteria at lower taxonomic levels [170], were not able to classify the main bacteria present in the vaginal microbiota of cows at the species level, either because of the poor quality of the databases or the presence of unknown organisms. This study highlights the need for more efforts to improve current databases.

In this context, the record of existing bacteria in a database is in the public domain and may not be updated as the nomenclature is refined over time. Therefore, closely related species described at different times may be assigned different taxonomies based on the best publishing practices [171,172]. Thus, uniquely identified genomes may be duplicated because they are registered with other names or accession numbers, thereby underestimating the number of bacteria in the sample. To reduce mapping limitations, the analyzed sequences can be compared to reference genomes and to each other to determine and verify whether the sample contains new taxa that are different from those found in other samples [172]. Many types of software have been developed to correct possible reading errors and recognize strains with exclusive genes. They have also enhanced the study of microbial communities, allowing researchers to accurately reconstruct sequenced communities at higher resolutions [173].

An essential factor is that identifying genes in the sample does not indicate that the microorganisms are a consistent part of the microbiota, as they may no longer be viable. In addition, amplicon sequencing does not allow for quantification, and results are expressed as a proportion of 100%. Thus, it is only possible to know if 20% of a particular species refers to one thousand or one million bacteria using quantitative methods, such as quantitative PCR [174]. Moreover, the fact that most species have a low relative abundance does not mean they have less influence on the host organism. Low-abundance intestinal bacterial communities contain genes responsible for critical metabolic processes for the microbial system, potentially triggering activities of the more dominant communities [175]. Thus, taxa with a low abundance or rarity may play essential roles in the vaginal microbiota of dairy cows in terms of reproduction, even if they remain unknown.

## 8. Future Perspectives

To date, microbiome research has led to extensive fundamental discoveries toward understanding the interaction of bacteria with the host and its surrounding environment. With the development of advanced technologies for sequencing genetic material, especially at the species level, situations or diagnoses that have remained unexplained for a long time have begun to be understood. In addition, it is crucial to update the available databases by sequencing and culturing so that they are accurate in identifying bacterial species and thus contain the correct taxonomies. More advanced data comparisons are necessary to ensure potential progress, which requires more harmonized and widely accepted standards and protocols.

Since interest in microbiota has expanded to several research groups, bacteria and their functions are currently being studied with different specificities. Metagenomic sequencing allows the tracking of bacterial populations, and its use as a predictive diagnostic tool is expected. It is believed that, in a few years, the reproductive potential or the risk of developing known pathologies in females can be determined through a quick test, thereby enabling preventive measures.

Furthermore, the possibility of identifying animals predisposed to reproductive disorders continues to be of interest to producers. Thus, removing them from the herd and minimizing economic losses while maintaining a more careful selection of dairy matrices will become possible. The key to further studies is to modulate bacterial communities by stimulating or introducing beneficial bacteria through probiotics and microbiota transplantation to promote reproductive efficiency and to identify those patterns predisposing the animals to diseases. Collectively, to provide more personalized and effective care, novel tools such as biological markers for good breeders or problem animals must be developed.

Therefore, future research must fully clarify the role of all variables, dependencies, and interactions among the microbiome, environment, hormonal influences, and the host, specifically for reproduction.

## 9. Conclusions

Identifying the likely causes of fertility problems and low pregnancy rates in cattle is a challenge for researchers. The complexity of the bacterial communities that constitute healthy microbiota contributes to this obstacle. Extensive research has examined microbiota, especially in the digestive tract, and has helped link dysbiosis to health problems. However, research on the composition of the vaginal microbiota remains limited. Therefore, the scientific community should focus on advancing databases of bacterial sequences at the species level in bovine species, as well as performing better and more targeted investigations of the reproductive microbiota in cows at different stages of life and production. Finally, this knowledge will assist in the effective use of bacterial communities for the treatment and prevention of the most diverse diseases that affect the reproductive tract of bovine females, in addition to possible manipulation or their use in bioactive/devices aimed at increasing female fertility. Finally, research must continue toward a precise strategy for manipulation and the identification of more effective methods to achieve and maintain an optimal balance in the microbiota.

## Data Availability

Data are contained within the article and Supplementary Materials.

## Supplementary Materials

The following supporting information can be downloaded at: https://www.mdpi.com/article/10.3390/ani14131971/s1, Table S1: Phylum and genera of vaginal microbiota reported in healthy female cattle; Table S2: Phyla and genera of uterine microbiota reported in healthy female cattle. References [13,19,27,59,79,81,83,86,87,88,91,92,106,107,108,109,168,176,177,178,179,180,181] are cited in the Supplementary Materials.
