# Supplementary material for: Updates and Current Challenges in Reproductive Microbiome: A Comparative Analysis between Cows and Women
Source: Animals (Basel). 2024 Jul 3;14(13):1971. doi: 10.3390/ani14131971 (PMC11240322; doi:10.3390/ani14131971)
Supplement: Supplementary file 1 [file animals-14-01971-s001.zip › animals-3003309-supplementary.pdf]

animals

Supplementary Materials

Table 1. Phylum and genera of vaginal microbiota reported in healthy female cattle.

Phylum	Genera
**Actinobacteria**	*Actinobacteria* [1–6]; *Atopobium* [7]; *Corynebacterium* [8]; *Trueperella* [9].
**Bacteroidota**	*Alistipes* [10,11]; *Bacteroides* [2–6,8,10–14]; *Porphyromonas* [12,15]; *Odoribacter* [7]; *Prevotella* [11]; *Rikinella* [11].
**Firmicutes**	*Aeribacillus* [10]; *Anaerofustis* [7]; *Clostridium* [5,7,10,11]; *Enterococcus* [16]; *Eubacterium* [10]; *Lactobacillus* [16,17]; *Oscillibacter* [11]; *Peptoniphilus* [9]; *Helcococcus* [9]; *Ruminococcus* [10]; *Staphylococcus* [16]; *Streptococcus* [11]; *Weissella* [17].
**Fusobacteriota**	*Fusobacterium* [1,12,15,18]; Sneathia [12].
**Proteobacteria**	*Anaerobiospirillum* [5]; *Escherichia* [16]; *Histophilus* [4,5]; *Patsteurella* [18]; *Succinivibrio* [5].
**Tenericutes**	*Mycoplasma* [7,15]; *Ureaplasma* [2,5,12,13,15].

Table 2. Phyla and genera of uterine microbiota reported in healthy female cattle.

Phylum	Genera
**Actinobacteria**	*Arcanobacterium* [19]; *Corynebacterium* [20]; *Trueperella* [20,21]; *Propionibacterium* [19].
**Bacteroidota**	*Bacteroides* [19]; *Parabacteroides* [19].
**Firmicutes**	*Agathobacter* [9]; *Anaerococcus* [19]; *Peptostreptococcus* [19]; *Staphylococcus* [20]; *Streptococcus* [20,22,23];.
**Fusobacteriota**	*Fusobacterium* [9,19]; Streptobacillus [9].
**Proteobacteria**	*Escherichia* [20,21]; Mannheimia [22]; *Patsteurella* [22].
**Tenericutes**	*Mycoplasma* [22]; *Ureaplasma* [19,22].

References

1. Miranda-CasoLuengo, R.; Lu, J.; Williams, E.J.; Miranda-CasoLuengo, A.A.; Carrington, S.D.; Evans, A.C.O.; Meijer, W.G. Delayed Differentiation of Vaginal and Uterine Microbiomes in Dairy Cows Developing Postpartum Endometritis. *PLoS One* **2019**, *14*, e0200974, doi:10.1371/JOURNAL.PONE.0200974.
2. Souza, A.K.; Zangirolamo, A.F.; Droher, R.G.; Bonato, F.G.C.; Alfieri, A.A.; da Costa, M.C.; Seneda, M.M. Investigation of the Vaginal Microbiota of Dairy Cows through Genetic Sequencing of Short (Illumina) and Long (PacBio) Reads and Associations with Gestational Status. *PLoS One* **2023**, *18*, e0290026, doi:10.1371/JOURNAL.PONE.0290026.
3. Ni, J.; Wang, J.; Zhao, K.; Chen, Y.; Xia, S.; Lai, S. Vaginal Microbiome Dynamics of Cows in Different Parities. *Animals* **2023**, *13*, 2880, doi:10.3390/ANI13182880/S1.
4. Quadros, D.L.; Zanella, R.; Bondan, C.; Zanella, G.C.; Facioli, F.L.; da Silva, A.N.; Zanella, E.L. Study of Vaginal Microbiota of Holstein Cows Submitted to an Estrus Synchronization Protocol with the Use of Intravaginal Progesterone Device. *Res Vet Sci* **2020**, *131*, 1–6, doi:10.1016/J.RVSC.2020.03.027.
5. Chen, S.Y.; Deng, F.; Zhang, M.; Jia, X.; Lai, S.J. Characterization of Vaginal Microbiota Associated with Pregnancy Outcomes of Artificial Insemination in Dairy Cows. *J Microbiol Biotechnol* **2020**, *30*, 804–810, doi:10.4014/JMB.2002.02010.
6. Giannattasio-Ferraz, S.; Laguardia-Nascimento, M.; Gasparini, M.R.; Leite, L.R.; Araujo, F.M.G.; de Matos Salim, A.C.; de Oliveira, A.P.; Nicoli, J.R.; de Oliveira, G.C.; da Fonseca, F.G.; et al. A Common Vaginal Microbiota Composition among Breeds of Bos Taurus Indicus (Gyr and Nellore). *Brazilian Journal of Microbiology* **2019**, *50*, 1115–1124, doi:10.1007/S42770-019-00120-3/METRICS.
7. Clemmons, B.A.; Reese, S.T.; Dantas, F.G.; Franco, G.A.; Smith, T.P.L.; Adeyosoye, O.I.; Pohler, K.G.; Myer, P.R. Vaginal and Uterine Bacterial Communities in Postpartum Lactating Cows. *Front Microbiol* **2017**, *8*, doi:10.3389/fmicb.2017.01047.
8. Amat, S.; Holman, D.B.; Schmidt, K.; Menezes, A.C.B.; Baumgaertner, F.; Winders, T.; Kirsch, J.D.; Liu, T.; Schwinghamer, T.D.; Sedivec, K.K.; et al. The Nasopharyngeal, Ruminal, and Vaginal Microbiota and the Core Taxa Shared across These Microbiomes in Virgin Yearling Heifers Exposed to Divergent in Utero Nutrition during Their First Trimester of Gestation and in Pregnant Beef Heifers in Response to Mineral Supplementation. *Microorganisms* **2021**, *9*, 2011, doi:10.3390/MICROORGANISMS9102011/S1.
9. Webb, E.M.; Holman, D.B.; Schmidt, K.N.; Pun, B.; Sedivec, K.K.; Hurlbert, J.L.; Bochantin, K.A.; Ward, A.K.; Dahlen, C.R.; Amat, S. Sequencing and Culture-Based Characterization of the Vaginal and Uterine Microbiota in Beef Cattle That Became Pregnant or Remained Open Following Artificial Insemination. *Microbiol Spectr* **2023**, *11*, doi:10.1128/SPECTRUM.02732-23/SUPPL_FILE/SPECTRUM.02732-23-S0001.DOCX.
10. Laguardia-Nascimento, M.; Branco, K.M.G.R.; Gasparini, M.R.; Giannattasio-Ferraz, S.; Leite, L.R.; Araujo, F.M.G.; De Matos Salim, A.C.; Nicoli, J.R.; De Oliveira, G.C.; Barbosa-Stancioli, E.F. Vaginal Microbiome Characterization of Nellore Cattle Using Metagenomic Analysis. *PLoS One* **2015**, *10*, e0143294, doi:10.1371/JOURNAL.PONE.0143294.
11. Rodrigues, N.F.; Kästle, J.; Coutinho, T.J.D.; Amorim, A.T.; Campos, G.B.; Santos, V.M.; Marques, L.M.; Timenetsky, J.; de Farias, S.T. Qualitative Analysis of the Vaginal Microbiota of Healthy Cattle and Cattle with Genital-Tract Disease. *Genetics and Molecular Research* **2015**, *14*, 6518–6528, doi:10.4238/2015.June.12.4.
12. Bicalho, M.L.S.; Lima, S.; Higgins, C.H.; Machado, V.S.; Lima, F.S.; Bicalho, R.C. Genetic and Functional Analysis of the Bovine Uterine Microbiota. Part II: Purulent Vaginal Discharge versus Healthy Cows. *J Dairy Sci* **2017**, *100*, 3863–3874, doi:10.3168/jds.2016-12061.

13. Deng, F.; McClure, M.; Rorie, R.; Wang, X.; Chai, J.; Wei, X.; Lai, S.; Zhao, J. The Vaginal and Fecal Microbiomes Are Related to Pregnancy Status in Beef Heifers. *J Anim Sci Biotechnol* **2019**, *10*, 1–13, doi:10.1186/S40104-019-0401-2/FIGURES/6.
14. Wang, Y.; Wang, J.; Li, H.; Fu, K.; Pang, B.; Yang, Y.; Liu, Y.; Tian, W.; Cao, R. Characterization of the Cervical Bacterial Community in Dairy Cows with Metritis and during Different Physiological Phases. *Theriogenology* **2018**, *108*, 306–313, doi:10.1016/j.theriogenology.2017.12.028.
15. Quereda, J.J.; Barba, M.; Mocé, M.L.; Gomis, J.; Jiménez-Trigos, E.; García-Muñoz, Á.; Gómez-Martín, Á.; González-Torres, P.; Carbonetto, B.; García-Roselló, E. Vaginal Microbiota Changes During Estrous Cycle in Dairy Heifers. *Front Vet Sci* **2020**, *7*, 552090, doi:10.3389/FVETS.2020.00371/BIBTEX.
16. Otero, C.; Saavedra, L.; Silva de Ruiz, C.; Wilde, O.; Holgado, A.R.; Nader-Macias, M.E. Vaginal Bacterial Microflora Modifications during the Growth of Healthy Cows. *Lett Appl Microbiol* **2000**, *31*, 251–254, doi:10.1046/j.1365-2672.2000.00809.x.
17. Wang, J.; Sun, C.; Liu, C.; Yang, Y.; Lu, W. Comparison of Vaginal Microbial Community Structure in Healthy and Endometritis Dairy Cows by PCR-DGGE and Real-Time PCR. *Anaerobe* **2016**, *38*, 1–6, doi:10.1016/J.ANAEROBE.2015.11.004.
18. Messman, R.D.; Contreras-Correa, Z.E.; Paz, H.A.; Perry, G.; Lemley, C.O. Vaginal Bacterial Community Composition and Concentrations of Estradiol at the Time of Artificial Insemination in Brangus Heifers. *J Anim Sci* **2020**, *98*, doi:10.1093/jas/skaa178.
19. Machado, V.S.; Oikonomou, G.; Bicalho, M.L.S.; Knauer, W.A.; Gilbert, R.; Bicalho, R.C. Investigation of Postpartum Dairy Cows' Uterine Microbial Diversity Using Metagenomic Pyrosequencing of the 16S RRNA Gene. *Vet Microbiol* **2012**, *159*, 460–469, doi:10.1016/J.VETMIC.2012.04.033.
20. Wagener, K.; Prunner, I.; Pothmann, H.; Drillich, M.; Ehling-Schulz, M. Diversity and Health Status Specific Fluctuations of Intrauterine Microbial Communities in Postpartum Dairy Cows. *Vet Microbiol* **2015**, *175*, 286–293, doi:10.1016/J.VETMIC.2014.11.017.
21. Prunner, I.; Pothmann, H.; Wagener, K.; Giuliodori, M.; Huber, J.; Ehling-Schulz, M.; Drillich, M. Dynamics of Bacteriologic and Cytologic Changes in the Uterus of Postpartum Dairy Cows. *Theriogenology* **2014**, *82*, 1316–1322, doi:10.1016/J.THERIOGENOLOGY.2014.08.018.
22. Santos, T.M.A.; Gilbert, R.O.; Bicalho, R.C. Metagenomic Analysis of the Uterine Bacterial Microbiota in Healthy and Metritic Postpartum Dairy Cows. *J Dairy Sci* **2011**, *94*, 291–302, doi:10.3168/jds.2010-3668.
23. Diaz-Lundahl, S.; Nørstebø, S.F.; Klem, T.B.; Gilfillan, G.D.; Dalland, M.; Gillund, P.; Krogenæs, A. The Microbiota of Uterine Biopsies, Cytobrush and Vaginal Swabs at Artificial Insemination in Norwegian Red Cows. *Theriogenology* **2023**, *209*, 115–125, doi:10.1016/J.THERIOGENOLOGY.2023.06.024.
